# Authentic Leadership and Employee Resilience: A Moderated Mediation Analysis

**DOI:** 10.3389/fpsyg.2022.901085

**Published:** 2022-07-01

**Authors:** Yanhui Mao, Yao Lai, Yuxi Zhai, Mei Xie, Junkai Yu, Qiutong Wang, Shaokai Lu, Jianhong Ma, Marino Bonaiuto

**Affiliations:** ^1^Department of Psychology and Behavioral Sciences, Zhejiang University, Hangzhou, China; ^2^Psychological Research and Counseling Center, Institute of Applied Psychology, Southwest Jiaotong University, Chengdu, China; ^3^School of Economics and Management, Southwest Jiaotong University, Chengdu, China; ^4^China Railway Construction Group Co., Ltd., Chengdu, China; ^5^School of Foreign Languages, Southwest Jiaotong University, Chengdu, China; ^6^School of Economics and Business Administration, Chongqing University, Chongqing China; ^7^Department of Psychology of Developmental and Social Processes, Sapienza University of Rome, Rome, Italy

**Keywords:** authentic leadership, organizational identification, employee resilience, flow, optimal experience

## Abstract

Authentic leadership is essential for predicting employee resilience. However, despite fruitful findings, more adapted models of authentic leadership – employee resilience based on empirical findings can serve as a guide to understand the complex mediators and moderators in different industries such as in construction engineering project organizations during the turbulent pandemic. This study, therefore, based on the organizational identification theory and flow theory through the lens of positive organizational psychology, aims to disentangle the authentic leadership—employee resilience association by investigating their underlying mechanism and their boundary condition. To test our hypothetical model, we applied a cross-sectional design with data collected from a large sample of 884 employees from a big enterprise in China. Findings from confirmatory factor analysis, structural equation modeling analysis, and Hayes’s conditional process model indicated that: authentic leadership positively predicted employee resilience through the partial mediation effect of organizational identification, and such a mediation model was moderated by the experience of flow. In other words, flow moderated the relationships between authentic leadership, organizational identification, and employee resilience. Findings provide evidence for cultivating leaders’ authenticity in promoting their subordinates’ resilience; findings also highlight the significance of organizational identification in bridging authentic leadership and employee resilience and the essential role of flow experience in supporting the relationships mentioned above.

## Introduction

Since December 2019, the highly contagious and fast-spreading novel coronavirus disease (COVID-19) has posed severe threats to individuals’ well-being, not only physically but also psychologically (Habersaat et al., 2020). To cope with such a universally tough challenge, people had to stay at home for a long time due to the prescribed strategies implemented by the government and discretionary self-isolation (Pagliaro et al., 2021; Yuan, 2021; Mao et al., 2022). This resulted in numerous companies shutting down and millions of employees losing their jobs. Facing such turbulent challenges, employees with higher levels of resilience could combat life stress and stay healthy, while those with lower levels of resilience confronted mental distress (Elisei et al., 2013). Therefore, we sought to explore how and when to build, maintain, and promote employees’ resilience at work to maintain their health and well-being during the COVID-19 pandemic. We formed a research group and randomly interviewed some employees in a central-government-owned construction enterprise in southwest China that ranked 47th on Fortune’s annual Global 500 list in 2021. According to their responses toward the understanding and experience of resilience, we summarized several commonly and mainly addressed keywords that might be the factors affecting employee resilience, such as authentic leadership (e.g., “*My leader delivers authenticity to me in my daily work”*), organizational identification (e.g., “*My organization’s success/failure is also my success/failure*”), and the universal human optimal experience of flow (“*I do this job because I enjoyed and loved this work, it is part of my important career*”).

Among these factors, leadership is considered one of the most influential factors affecting an employee’s resilience. It fosters a supportive organizational context for employees (Ou et al., 2014) and benefits employee growth and development by reinforcing organizational learning (Owens and Hekman, 2012). Standing on the social exchange theory (SET; Blau, 1964), higher levels of leadership correlate with higher levels of information sharing and perceived psychological safety (Hu et al., 2018). Luthans and Avolio (2003) defined authentic leadership as “a process that draws from both positive psychological capacities and a highly developed organizational context, which results in both greater self-awareness and self-regulated positive behaviors on the part of leaders and associates, fostering positive self-development” (p. 243). Authentic leadership, as a subordinate leadership theory and one of the leadership styles (Avolio, 2005; Walumbwa et al., 2008), contributes to employees’ feeling more likely to be supported and perceiving themselves as insiders of an organization due to perceived authenticity (Avolio et al., 2004). However, to the best of our knowledge, the study of authentic leadership concerning employee resilience among the population of Chinese employees—especially in light of the prevailing COVID-19, has not yet been fully addressed.

Empirical evidence has also found that authentic leadership is positively associated with employees’ affective attachment to and cognitive identification with their organizations (Leroy et al., 2012). Perceived insider identification, or organizational identification, is a product of employees’ cognitive processes derived from social interaction; it is also the process whereby an employee feels himself in an important organization and is critical to view one’s organizational membership in a favorable light (Schaubroeck et al., 2017). Organizational identification has been proven to fully mediate the association between perceived organizational support and organizational citizenship behavior (Shen et al., 2014) and between perceptions of organizational context and job attitudes among Chinese subordinates (Ngo et al., 2013). The stronger the employees’ identification with their organizations, the more ready they are to solve problems confronted at work, and the stronger their resilience in coping with challenges (Kodama and Tokaji, 2010).

Among other predictive factors, flow is a holistic affective and cognitive state that occurs when employees perform daily work activities at hand at full capacity, with full concentration, complete involvement, loss of self-consciousness, and optimal enjoyment (Csikszentmihalyi, 1975). Positive psychological functioning like flow experienced at work further fosters creative and innovative outputs with an immense stance of proficiency and effectiveness (Ishimura and Kodama, 2006) since flow is positively associated with psychological resilience and well-being at the workplace (Peterson et al., 2005; Mao et al., 2016; Wu et al., 2021).

Prior research on resilience concerning authentic leadership has received significant attention (Rego et al., 2012; Liu et al., 2015). Some studies have proved that authentic leadership influences resilience (Gouldner, 1960). Some found that authentic leadership activates employee resilience during the organizational crisis and influences resilience through organizational resilience (Teo et al., 2017). Still, some demonstrated that leadership could directly and significantly influence job satisfaction through the influence of resilience (Glallonardo et al., 2010; Wong and Laschinger, 2013). Work-related flow experience affects organizational commitment and work behavior (Kim et al., 2019). Additionally, it has been proved that organizational identification can predict work engagement through psychological resilience (Lyu et al., 2020). Therefore, during such a turbulent COVID-19 contingency that posed severe challenges to construction engineering enterprises, the present study attempts to respond to the need identified by Kuntz et al. (2016) and Koni et al. (2019) to explore further the relations between psychological states (flow), social identity (e.g., organizational identification) and adaptive capabilities (e.g., employee resilience) among Chinese frontline construction employees, aiming to address the gap and to explore the authentic leadership-employee resilience relationship by elucidating their underlying mechanism and boundary condition taking into consideration of organizational identification and flow experience at work, and provide practical suggestions for construction engineering managers in China and the world at large.

Globally, the construction engineering industry is regarded as one of the most critical sectors, contributing to a country’s GDP and the success of megaprojects in developed and developing countries. To this end, these enterprises are working hard to get a competitive edge by cultivating resilient employees who can operate effectively and efficiently under enormous pressure (Sarwar et al., 2017). Regionally, and specifically, in China, construction engineering project organizations, unlike regular enterprises, confront many challenges (e.g., constrained within transactional cost and construction cycles, pushing deadlines, the complexity of construction sites, poor living conditions, the one-time organization that is based on a project by project, and so forth), Yang et al. (2021) propose that employees psychological resilience is highly demanded. However, there is limited empirical evidence on the nature of psychological resilience with its impact on performance in this industry in China (He et al., 2019). To date, most relevant findings have been focused on the healthcare industry (Glallonardo et al., 2010; Wong and Laschinger, 2013; Lyu et al., 2020; Labrague, 2021), but hardly any research focusing on working employees from construction engineering projects organizations. This research is meant for construction companies since it shows that focusing on the development of psychological resilience may aid with construction engineering project success. Furthermore, authentic leadership may be a critical stimulant in completing a construction engineering project. As a result, the present study was designed with this backdrop.

## Theoretical Framework and Hypotheses

### Authentic Leadership and Employee Resilience

Within positive organizational psychology, authentic leadership is a leadership style that combines both positive psychological abilities and a highly developed organizational context to foster positive behaviors in leaders and associates; it produces self-development in everyone (Avolio et al., 2004). Authentic leadership, among scholars and in consensus, is manifested by four dimensions (Avolio et al., 2004; Luthans et al., 2005; Walumbwa et al., 2008): (1) self-awareness (i.e., a realization of one’s strengths and weaknesses, as well as one’s worldview), (2) relational transparency (i.e., exhibiting one’s authentic self by publicly sharing information and feelings when appropriate), (3) internalized moral perspective (i.e., self-governing one’s behavior guided by internal moral standards), and (4) balanced processing (i.e., having an objective analysis of relevant information/data before making decisions). Given that authentic leaders are not only denoted as morally “*good*” due to their authenticity and sincerity, they are also very effective leaders capable of triggering superior organizational outcomes. Thus, authentic leadership has been proclaimed the catalyst to organizational performance and economic growth and heralded as an answer to a broad range of ethical and environmental challenges (Alvesson and Sveningsson, 2013). Specifically, due to the sudden widespread shift to home-based teleworking and other detrimental impacts of COVID-19 on employees in the construction engineering project organizations, the importance of authentic leaders is of great significance in developing and maintaining employee resilience.

To cope with such challenging circumstances, employees will also need to develop salient personal capabilities to exploit organizational resources to adapt to adversity and flourish at work continually (Lengnick-Hall et al., 2011; Kuntz et al., 2016). To this end, employee resilience, in the limelight among scholars and practitioners (Näswall et al., 2019), has been promoted as essential for successful organizational functioning, such as inter-organizational cooperation, organizational learning, knowledge sharing, and organizational flexibility (Jozaei and Mitchell, 2018; Yang et al., 2021). Put another way, employee resilience is of great significance for supporting an organization’s short-term survival and long-term sustainable growth and an employee’s self-sustainable development (Cooper et al., 2019; Isidro and Calleja, 2021).

Numerous research has already proposed the positive impact of authentic leadership on employee resilience in organizational settings (Rego et al., 2012; Liu et al., 2015; Abd-El Aliem and Abou Hashish, 2021) because authentic leaders can give employees support when needed, thereby enhancing the resilience of employees by (1) helping employees recover from adversity, (2) not only withstanding but also thriving in the face of high-level positive changes since authentic leaders have a high degree of awe of potential hazards and they can formulate contingency plans to bring employees’ hope, confidence, optimism, etc. (Gardner and Schermerhorn, 2004). Additionally, according to the principle of reciprocity (Gouldner, 1960) and social exchange theory, employees are more likely to engage in resilient reactions in organizational crisis when authentic leaders ensure their perceived support, exchange quality, affective commitment, trust, and psychological contract fulfillment (Meng et al., 2019; Zhu et al., 2019). Taken together, we wish to confirm if such a relationship also happens in extreme situations of COVID-19 that resilience is highly demanded, especially for the employees from the construction engineering project organizations as the whole industry is challenged; therefore, we propose that:

***H1:*** Authentic leadership perceived by employees is positively associated with their resilience.

### Authentic Leadership and Employee Resilience: The Role of Organizational Identification

Organizational identification is defined as the perception of oneness or belongingness to an organization or membership in an organization (Ashforth and Mael, 1989). As members would possess both a rational sense of contract and responsibility in the organization, as well as an irrational sense of belongingness and dependency, organizational identification is also a component of one’s social identity whereby a person comes to view him/herself as a member of a particular social entity within the organization (Dutton et al., 1994; Bergami and Bagozzi, 2000; Lapalme et al., 2009). Multiple studies conducted within general corporations have found that employees’ organizational identification is affected by their attitudes and behaviors under the influence of their leaders (Pellegrini and Scandura, 2008; Ravasi and Phillips, 2011). As leaders help facilitate the shaping of employees’ perceptions of social status within the organization (Lapalme et al., 2009) and further influence employees’ subsequent workplace behavioral reactions (Tyler and Blader, 2000), authentic leadership has been given credit for shaping organizational behaviors and employees’ work attitudes (Neider and Schriesheim, 2011). Meanwhile, employees who show personal identification with authentic leaders would dedicate internalized moral behaviors (Cui et al., 2021). Lyu et al. (2020) have indicated that identification with a leader who works in a hospital’s ward organization is the source of the identification with that ward organization. Therefore, they suggest cultivating and developing leaders’ authentic behaviors to promote organizational identification. Organizational identification mediates the relationship between authentic leadership and employees’ innovative behavior (Niu et al., 2018). Authentic leaders help new employees identify with the leader and the organization, and the identification boosts new employees’ self-efficacy in occupational coping (Fallatah et al., 2017). Personal identification can mediate the relationship between authentic leadership and internal whistleblowing (Liu et al., 2015). As organizational identification encourages employees to act more actively in challenging situations (i.e., COVID-19), employees’ performance is always supervised, evaluated, and affected by their leaders, it is reasonable to assume that a solid, authentic leadership may contribute to predicting employee resilience through organizational identification. Accordingly, we believe that:

***H2:*** Organizational identification mediates the relationship between authentic leadership and employee resilience.

### Authentic Leadership and Employee Resilience: The Role of Flow Experience

Prior studies have suggested that people with higher levels of resilience are inclined to engage in more positive and adaptive behaviors in adverse life events (Peng et al., 2012) and organizational crises (Teo et al., 2017). Prior work also suggested that a strong organizational identification held within the nurse population positively predicts their work engagement (Lyu et al., 2020), which to Seligman (2002), is the optimal human functioning experience at workflow.

Flow (Csikszentmihalyi, 1975) depicts a state of consciousness that when one is totally immersed in the working task or activity that is drastically enjoyable and rewarding, employees know how well they are doing since flow experience provides instant feedback not contingent upon any external reward but rather derives from the intrinsic motivation. Employees experiencing flow receive confirmation that their actions result in progress, leading to a sense of competence in the present and being pleased with their decision to pursue job activities (Csikszentmihalyi and LeFevre, 1989). To be in flow, employees should be intrinsically motivated to engage in work and attain a match between the challenge presented and their corresponding skills (Csikszentmihalyi, 2000). Since the combinations of high challenges-high skills situations are mostly found at work in everyday human life, flow is perceived more frequently and intensely (Mao et al., 2016). When a working activity provides the opportunity for an employee’s skills to be leveraged as a springboard and refined to the utmost, the work itself becomes more interesting and enjoyable, and employees also become more productive (Luthans et al., 2007; Peifer and Zipp, 2019).

The significant positive association between authentic leadership and work-related flow can be traced in many studies (Zubair and Kamal, 2015) since authentic leaders are more likely to inspire and encourage people via increased internal stimulation and intrinsic enthusiasm, as well as an increased experience of positive emotions such as hope and optimism (Ilies et al., 2005). Besides, numerous studies have elucidated that experiencing flow is positively associated with a series of psychological capital such as resilience, efficacy, sense of fulfillment, and positive affect (Schmidt, 2003; Asakawa, 2010; Mao et al., 2020b; Cui et al., 2021). According to the broaden-and-build theory, the positive effect from flow creates positive spirals that create further positive experiences, thoughts, and feelings that promote optimal functioning and well-being (Fredrickson, 2001). Flow is a positive affective and cognitive experience that is widely reported irrespective of culture, age, or gender (Csikszentmihalyi, 1975; Wu et al., 2021), as well as in the workplace (Ignjatovic et al., 2022). Higher levels of flow experience are associated with stronger identity experiences, such as place identity, organizational identity, and community identity (Bonaiuto et al., 2016; Mao et al., 2016, 2022). And intrinsic motivation is the essence of flow for promoting performance in specific work situations which results in experiences of pleasure, enjoyment, and satisfaction in working tasks (Deci and Ryan, 1985). We may safely assume that as long as there is flow being experienced, one may experience reinforced identification toward one’s organization, and may exhibit strong resilience in coping with various challenges such as COVID-19, since optimal enjoyment or fun is a crucial factor of flow that can promote everyday work engagement instead of job quit (Christandl et al., 2018).

Flow can be considered as both a trait and state variable, still, almost no research has considered its moderation effect, since scholars lack a distinction between the antecedents, characteristics, and consequences of flow and flow itself (Engeser and Rheinberg, 2008). However, we can trace some implicit evidence of its potential moderating effect. For example, as one core element of flow, enjoyment has been proven to have significant moderating effects on the relationship between people’s interaction and learning persistence (Yu et al., 2020). It is also verified that work engagement (flow) has a moderation role in people’s self-efficacy and, in turn, their well-being (Huang et al., 2022). As one prerequisite of flow, trait intrinsic motivation is shown to be the most potent moderator of the flow model of the state of intrinsic motivation in the Chinese population (Moneta, 2004). To this end, our study was the first to try to fill in the research gap regarding flow as a moderator, not to mention the relationship between authentic leadership and employee resilience.

**FIGURE 1:**
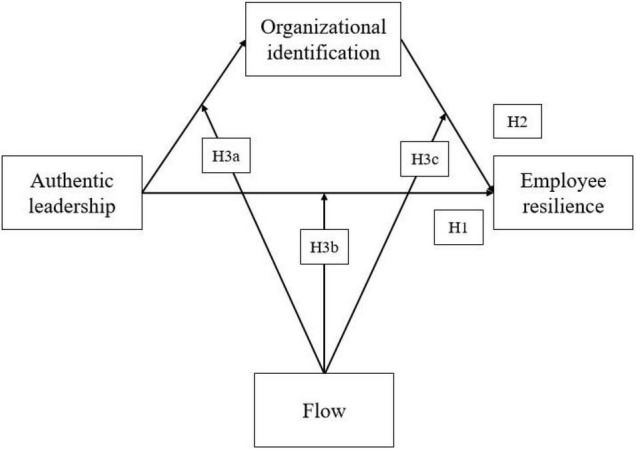
The conceptual framework.

Considering the positive human flourishing experience of flow, we sought to explore its boundary condition within the relationship among authentic leadership, organizational identification, and employee resilience (see Figure 1). Since all the relationships and performance depend on whether or not employees work happily, feel meaningful, and match skills and challenges at their workplace.

***H3a:*** Flow moderates the relationship between authentic leadership and employee resilience.
***H3b:*** Flow moderates the relationship between authentic leadership and organizational identification (innovative hypothesis).
***H3c:*** Flow moderates the relationship between organizational identification and employee resilience.

## Materials and Methods

### Participants and Procedure

We approached 900 frontline employees working on a full-time basis at a central government-owned construction engineering project organization. We chose this enterprise as the research site not only due to its location in the same city for convenient data collection, but more importantly, because of its management strategies—it always strives to hear the voice of the employees, cares about their workflow and well-being, and aims to increase their sense of gain, happiness and security, thereby enhancing the employees’ resilience and construction project quality. The participants were recruited in a cross-sectional survey with the consent of voluntary participation. We did the pilot test before formally administering the questionnaire survey to ensure the clarity of each item. In the questionnaire administration, we excluded the inattentive responses. For instance, some response answers were always “3” instead of ranging from “1” to “5,” and some responses failed on attentional check questions. After deleting those with the inattentive response falling from the attentional check, we retained 884 frontline employees as our final sample valid for further data analysis. Among them, 224 were women, and 640 were men (aged 18–60 years, Mean = 32.35, *SD* = 0.857), with 62.2% of them having worked in their companies for more than 3 years. It was worth noting that data from the present work were a part of a larger project that aimed to evaluate Authentic Leadership and Employee Well-being (Mao et al., in press).

As for the procedure, first of all, we translated the original English questionnaire items followed by Brislin’s (1970) back-translation procedure. We then created an online Chinese version questionnaire administered for a pilot test to assure that each item has the potential to tap into the same target construct in both the original English version and the Chinese language version (Beins, 2013). Subsequently, we approached the organizational employees with a generated QR code of the validated Chinese language questionnaire via the *WeChat* group (an instant messaging system popular in China), primarily established by HR managers and then project managers. Our participants were recruited by their project managers; they were notified that the survey was anonymous, and the participation was voluntary. They could withdraw at any time during the survey process, and their response data would be for scientific purposes only. Participants’ informed consent was obtained before answering the online survey question. The local ethical committee approved the study at the first author’s university. The survey was administered with self-reports based on employees’ interaction with their organizational project leaders, referring to their daily work experience recalling the past 6 months when the COVID-19 pandemic was massively present. The questionnaire survey took approximately 5–10 min to complete, and data were collected in December 2020.

### Measures

All measures (except flow) presented in this study were based on a 5-point Likert type scale ranging from 1 (strongly disagree) to 5 (strongly agree).

#### Authentic Leadership

An employee’s perceived authentic leadership was measured by a 16-item Authentic Leadership Questionnaire (ALQ; Walumbwa et al., 2008) comprising four dimensions, respectively, on Self-Awareness (e.g., “*My vertical leader seeks feedback to improve interactions with others*.” Cronbach’s *α = 0.931*), Relational Transparency (e.g., “*My vertical leader solicits views that challenge his or her deeply held positions*.” Cronbach’s *α = 0.943*), Internalized Moral Perspective (e.g., “*My vertical leader tells me the hard truth*.” Cronbach’s *α = 0.933*), and Balanced Processing (e.g., “*My vertical leader analyses relevant data before coming to a decision*.” Cronbach’s *α = 0.939*). This scale has been widely used (Cheng et al., 2020; Norouzinia et al., 2020), and the Cronbach’s α for the whole scale was 0.980. Considering there are too many items of authentic leadership that may influence the accuracy of the structural model (e.g., chi-square, degree of freedom ratio, comparative fit index, etc.), we treated these four dimensions as one dimension in model testing, drawing on Jang and Chen’ research methods Jang and Chen (2022).

#### Organizational Identification

Organizational identification was tested with a 6-item scale originated by Mael and Ashforth (1992). Sample items were “*When someone criticizes my company, it feels like a personal insult*”; “*I am very interested in what others think about my company.*” Higher sum scores indicated greater levels of organizational identification. The internal consistency of this scale was reported high in previous studies (e.g., Mao et al., 2016; Wang et al., 2017), while Cronbach’s alpha regarding the present sample was 0.861.

#### Employee Resilience

Employee resilience was assessed by a 9-item Employee Resilience Scale (EmpRes) developed by Näswall et al. (2015). Sample items were “*I transform change at work into an opportunity for growth*” and “*I re-evaluate my performance and continually improve the way I do my work*.” This scale has been validated in the Chinese cultural context (Cronbach’s α = 0.85; Zhu et al., 2019). The Cronbach’s alpha concerning the present sample was 0.949.

#### Flow

Flow was examined using a 7-item scale adapted from the Swedish Flow Proneness Questionnaire (SFPQ; Ullén et al., 2012) that pertained to the flow frequency at a work domain. The sample item was “*I have a clear picture of what I want to achieve, and what I need to do to get there?*” Such a scale has been validated globally with good reliability and validity (De Manzano et al., 2013; Liu and Csikszentmihalyi, 2020; Mao et al., 2020a,b; Wu et al., 2021). However, in the present work, one item (“*I feel bored when I do something at work*”) with low factor loading was deleted to improve its construct validity and reliability. In this study, the remaining six items, SFPQ also possessed good internal consistency with its Cronbach’s alpha value of 0.813.

### Data Analytic Strategy

First, descriptive statistics, correlational indices, Cronbach’s alpha, and Kaiser–Meyer–Olkin (KMO) were tested via SPSS (25.0). When analyzing the adjustment model, SPSS was used to define the calculation method of the latent variable as the sum of the observed indicators corresponding to the latent variables. Since the SPSS adjustment model only supports observed indicators, we then calculated the average (that is, converted the latent variable into the observed variable). Second, confirmatory factor analysis (CFA) and structural equation modeling (SEM) was conducted through AMOS (21.0). The hypothesized mediation model (OI mediates the relationship between AL and ER) was examined using AMOS’s Maximum Likelihood Estimation approach. Model fit indices as suggested by Hu and Bentler (1999) were considered as follows: Chi-square and degree of freedom ratio (χ^2^/df < 3), comparative fit index (CFI > 0.90), normative fit index (NFI > 0.90), goodness-of-fit index (GFI > 0.90), incremental fit index (IFI > 0.90), and the root means square of approximation (RMSEA < 0.08). Finally, the proposed flow moderation (see Figure 1) was assessed using Hayes’s (2012) Macro Process Model (No. 59) plugged in SPSS.

## Results

### Inspection of Common Method Biases

As our data were one-time, single source, and cross-sectional that may be subject to CMB, we separated similar items by numbering the questionnaire items at the questionnaire design stage and appropriately modified response statements for items of different variables. Besides, we applied Harman single factor test to check whether CMB was serious. As the yielded results were far below the cut-off value (the variance explained by the first factor without rotation was below 50%, Podsakoff and Organ, 1986; CMB presents a substantial potential for upward bias in relationships only when variance was above 70%, Fuller et al., 2016), therefore, there was no severe CMB.

#### Descriptive Statistics and the Validity Test

Table 1 provides the means, standard deviations, and correlations of all the study variables. As expected, there were significant and positive correlations between authentic leadership, organizational identification, employee resilience, and flow. All participants were from the same company, and their educational background and income were more or less at the same level without any significant differences. We, therefore, included only age and gender as our control variables.

**TABLE 1:** Descriptive statistics and correlations.

Variable	Mean	*SD*	1	2	3	4	5	6
1. AL	3.78	0.77	1					
2. OI	3.76	0.78	0.58**	1				
3. ER	3.90	0.64	0.64**	0.53	1			
4. Flow	3.57	0.70	0.39	0.41	0.54	1		
5. Age	32.35	0.86	–0.04	–0.03	0.01	0.04	1	
6. Gender	1.28	0.45	−0.14**	−0.14**	–0.06	–0.05	0.16834**	1

*AL, authentic leadership; OI, organizational identification; ER, employee resilience.*

**p<0.01, two-tailed.

To check the reliability and validity of the questionnaire, convergent validity was confirmed by the following three criteria: (1) all path weights yielded from observed indicators to latent variables were significant (*p* < 0.001); (2) the values of composite reliability (CR) regarding all latent variables ranged from 0.779 to 0.970, meeting the minimum criteria of 0.70 (Nunnally and Bernstein, 1994); and (3) the values of the average variance extracted (AVE) ranged from 0.540 to 0.896, which was greater than the threshold value of 0.50. It was indicated that all latent variables were represented by their respective observed indicators.

Table 2 presents the results of KMO and the Bartley spherical test. As indicated, the KMO value for authentic leadership, organizational identification, employee resilience, and flow was above 0.7 (*p* < 0.001), and the value for the Bartley spherical test was below the 0.01 levels. To this end, the KMO value and the Bartley spherical test indicated that the measurement model had good structural validity and was suitable for further confirmatory factor analysis (CFA).

**TABLE 2:** Validity and reliability of study variables.

Construct	KMO	Bartlett’s test	Cronbach’s α	AVE	CR
AL	0.980	0.000	0.980	0.666	0.970
OI	0.879	0.000	0.861	0.896	0.846
ER	0.946	0.000	0.949	0.566	0.921
Flow	0.797	0.000	0.813	0.540	0.779

### Test of the Measurement Model

We then performed CFA within AMOS to validate the measurement model. As indicated, the measurement model that composed of four latent variables (AL, ER, OI, and Flow) with corresponding 37 observed indicators revealed a satisfactory fit to our data: χ^2^/df = 2.74, *p* < 0.001; RMSEA = 0.044; CFI = 0.965; GFI = 0.977; NFI = 0.946. Therefore, all latent variables were represented by their respective observed indicators.

### Test of Structural Model: Organizational Identification Mediates the Relationship Between Authentic Leadership and Employee Resilience

**FIGURE 2:**
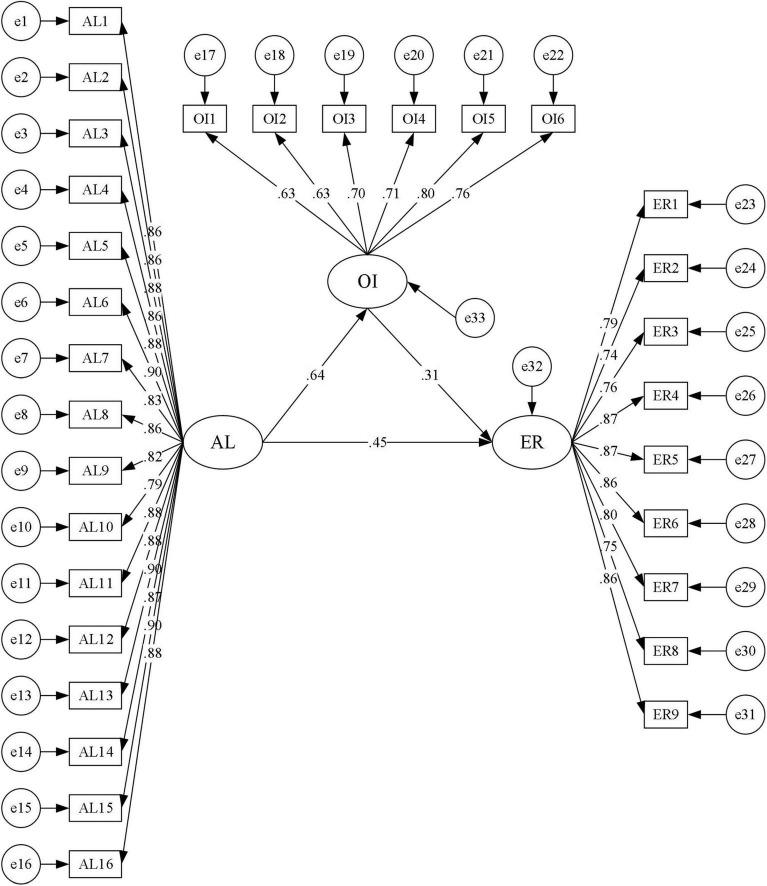
Test of the mediation model.

Structural equation modeling (SEM) via the maximum likelihood (ML) estimation method was applied to examine the hypothesized mediation model (driven by theory and literature). Results of SEM (see Figure 2) indicated good fit according to a set of fitting criteria: χ^2^/df = 2.628, *p* < 0.001; CFI = 0.976; NFI = 0.962; and RMSEA = 0.043.

The standardized path coefficient between authentic leadership and employee resilience is presented in Table 3, which indicates a significant and positive direct effect of authentic leadership on employee resilience (*p* < 0.01), supporting our hypothesis **H1**. When organizational identification was entered into the model, such a direct effect was still significant (*p* < 0.01). Followed that, the bootstrap estimation procedure (5000 bootstrap samples) was applied to test the indirect effect of authentic leadership on employee resilience through organizational identification. As zero was not included within 95% confidence intervals (CI), it yielded a significant indirect effect. Taken together, the direct effect (0.453) and the mediation effect (0.198) accounted for 69.59 and 30.41% of the total effect (0.651), respectively. Therefore, our hypothesis **H2** was supported as a partial mediation model was confirmed.

**TABLE 3:** Standardized direct and indirect effects with 95% confidence intervals.

Hypotheses	Path	Standard-estimate	Bias-corrected 95% CI		*p*	Decisions
			Lower	Upper		
H1	AL-ER	0.453	0.356	0.548	0.001	Supported
H2	AL-OI-ER	0.198	0.141	0.265	0.001	Supported

#### Test of Moderation

The moderation effect was tested via Hayes’s Macro Process Model (2012) by controlling gender and age. As indicated in Table 4, when flow was entered in the model, the path of flow AL had significant predictive effect on OI (β = 0.088, *t* = −3.050, *p* < 0.01), as well as on ER (β = −0.109, *t* = −3.896, *p* < 0.001). Besides, the path of Flow to OI had a significant predictive effect on ER (β = 0.059, *t* = 2.262, *p* < 0.05), indicating that flow also moderated the predictive effect of OI on ER.

**TABLE 4:** Conditional process analysis.

Dependent variable	Predicting variable	Fitting indices			Coefficient	
		*R*	R^2^	*F*(*df*)	β	*t*
OI		0.6193	0.3836	109.2699		
	Gender				–0.110	−2.328**
	Age				–0.015	–0.601
	*AL*				0.478	15.995***
	*Flow*				0.235	7.303***
	*AL × Flow*				0.088	−3.050**
ER		0.7277	0.5295	140.8253		
	Gender				0.049	1.446
	Age				0.010	0.560
	*AL*				0.345	14.203***
	*OI*				0.135	5.588***
	*Flow*				0.282	11.672***
	*AL × Flow*				–0.109	−3.896***
	*OI × Flow*				0.059	2.262*

*p<0.05, **p<0.01, ***p<0.001.

**FIGURE 3:**
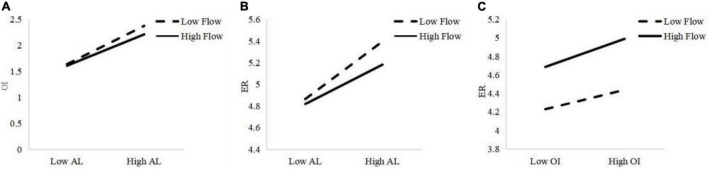
**(A)** Flow’s moderation effect on the path between AL and OI. **(B)** Flow’s moderation effect on the path between AL and ER. **(C)** Flow’s moderation effect on the path between OI and ER.

In terms of further simple slope analysis, for participants who experienced a lower level of flow, their perceived AL would have a significant positive predictive effect on OI (simple slope = 0.737), and such effect was stronger than participants who experienced a higher level of flow (simple slope = 0.602). This suggested that: with the increase in perceived flow, the predictive effect of AL on OI decreased gradually (see Figure 3A). Therefore, our hypothesis H3a was confirmed. A similar trend was also found, as indicated in Figure 3B. For participants who experienced a lower level of flow, their perceived AL would have a significant positive predictive effect on ER (simple slope = 0.531), and such an effect was stronger compared with participants who experienced a higher level of flow (simple slope = 0.364). That being said: with the increase of perceived flow level, the predictive effect of AL on ER also gradually decreased, which also confirmed hypothesis H3b.

However, the results of simple slope analysis demonstrated in Figure 3C suggested that: for those subjects who experienced a lower level of flow, though their perceived OI would have a significant positive predictive effect on ER (simple slope = 0.211), such a predictive effect was weaker in comparison to the impact on participants who experienced a higher level of flow (simple slope = 0.303). This indicated that: with the increase of perceived flow level, the predictive effect of OI on ER was gradually reinforced, therefore, confirming H3c.

## Discussion

### Major Findings

Leaders increasingly realize the value of positivity and focus on cultivating employee strengths rather than lingering on employee vulnerabilities and weaknesses (Avolio et al., 2004; Luthans et al., 2005). The present work sought to explore how and when authentic leaders could predict their employees’ resilience, especially in the current ongoing COVID-19 challenging environment. From a sample of 884 employees of a construction engineering project enterprise, we found that authentic leaders positively predict their followers’ resilience by increasing follower identification with the organization, such a partial mediation is moderated by the universal positive human experience of flow. Our findings support all the proposed hypotheses, with discussions provided as follows.

First of all, authentic leadership is essential for predicting employee resilience in a population from the construction engineering project organization. We found that authentic leadership was positively and directly correlated with employee resilience, in line with the principle of reciprocity (Gouldner, 1960), social exchange theory (SET; Blau, 1964), and considerable previous research (Rego et al., 2012; Liu et al., 2015; Zhu et al., 2019). This is easy to understand in that when employees perceive their leaders’ authenticity; they will feel more being trusted (Mao et al., 2022), viewing their role as being more significant so that they will exhibit more resilience by engaging in and coping with encountered challenges posed to their organizations.

Secondly, consistent with the previous study, we found that authentic leadership plays an underpinning role in increasing employee’s identification with their organizations (Pellegrini and Scandura, 2008; Ravasi and Phillips, 2011), and organizational identification plays a partial mediation role in the relationship between authentic leadership and employee resilience (Tyler and Blader, 2000; Ravasi and Phillips, 2011; Lyu et al., 2020). As an authentic leader is on behalf of his/her organization’s image, the more sincerity and authenticity one shows to one’s employee, building on the social exchange theory, the more this employee feels the leader is trustworthy and will appreciate the sincerity of their organization. The sense of belongingness to the organization, therefore, will become stronger. As for the partial effect of organizational identification, we could speculate on one of the possible explanations from the theory of social identity. In the case that organization members begin to have a sense of identity targeted at the organization to which they belong, initiative depersonalization behaviors would occur in organization members who realize that they share the organization’s fate (Turner et al., 1987; Vieru and Rivard, 2014), and they would become fixated on recovering.

Finally, the negative moderation that flow exerts on the links between authentic leadership and employee resilience and between authentic leadership and organizational identification can be interpreted as intrinsic motivation. Employees who have higher levels of flow experience at work are intrinsically motivated to work and therefore tend to be highly resilient, so they do not rely that much authenticity on leaders to build their resilience (nor the organizational identification partial mediation on that effect), ascribed to that they are already resilient thanks to flow. On the contrary, without flow experience, they can be resilient only if there are external, contextual conditions that facilitate them to achieve resilience. In fact, only the employees with lower flow levels can benefit from the virtuous chain from authentic leadership to employee resilience and chain from authentic leadership to organizational identification. These findings also imply that the highly desirable state of flow, which is connected with highly effective behaviors and high experience quality, may bring side effects. This is consistent with Csikszentmihalyi’s (1990) statements that enjoyable activities that produce flow have a potentially negative aspect: while they can improve the quality of existence by establishing order in the mind, they can also become addictive (such as workaholics), at which point the self becomes captive to a particular kind of order and thus unable to cope with life’s ambiguities (Csikszentmihalyi, 1990, p. 62). However, flow positively catalyzes organizational identification’s effect on employee resilience. No matter how much sense of organizational identification employees have, the more flow they can produce when they are working, the more resilience they can possess in challenging situations, which accords with the finding that flow is strongly linked with many positive individuals and organizational outcomes in workplace settings (Peters et al., 2014; Koni et al., 2019). The explanation of these moderating effects can also be attributed to the fact that flow is an intrinsically motivating (i.e., “autotelic”) psychological state of complete absorption in any job activity that can occur when one performs a task. Self-reported flow is linked to deviations from anticipated performance (Palomäki et al., 2021).

In a word, the findings are illuminating in that they demonstrate how authentic leadership contributes to the development and psychological empowerment of an employee’s resilience and that psychological capital can be developed both specifically through authentic leadership and, more broadly, through organizational belongingness. The findings shed light on how authentic leadership has the potential to calibrate employees’ psychological capital through a customized flow intervention program that is aligned with authentic leadership and perceived organizational identification.

### Implications

#### Theoretical Contribution

This study contributes to a more comprehensive understanding of authentic leadership’s impact on employees’ resilience through the lens of organizational identification and flow theory embedded in a positive organizational psychological perspective, such a relationship, however, has not yet been addressed in the available literature. Findings generated from a large sample of Chinese organizational employees are promising particularly in light of the fact that scholars of positive organizational behavior have urged researchers to investigate how followers’ PsyCap might be developed (e.g., Luthans et al., 2007). The framework linking authentic leadership and employee resilience that we proposed in this article contributes to the theoretical development of employee resilience. Given that organizational scholars and practitioners have recently shown great interest in the role of leadership in the workplace, this study advances authentic leadership theory in providing further evidence of the mechanisms by which authentic leaders affect follower PsyCap based on social exchange theory (Gouldner, 1960; Pellegrini and Scandura, 2008; Ravasi and Phillips, 2011), as well as extends the existing employee resilience theory in the sample employees from construction engineering project organization, characterized by cultivating its employees who tend to have great loyalty to supervisors and support the organizational goals. In particular, this study makes a novel contribution by analyzing the critical partial mediation effect of organizational identification, providing initial support for the theoretical arguments that organizational identification is essential to explain the authentic leadership-employee resilience linkage, as well as may have stronger implications for the link in collectivist countries than in individualistic ones.

Moreover, our results show that many variables’ impact on employee resilience may be conditional, suggesting that models of resilience may have to be revised to consider moderating factors. The present findings add further relevant insights to broaden-and-build theory and work-related flow theory by highlighting the critical moderator—flow experiences in comprehending different leadership effects and resilience outcomes, emphasizing the positive interaction between organizational identification and the flow experience. In a word, this is the first study to establish linkages between the four variables. We hope these efforts will spur conceptual work toward the development of a holistic paradigm of authentic leadership-employee resilience relationship with the contribution of organizational identification and flow.

#### Practical Implications

Our findings offer a number of practical implications for construction engineering project organizations**. First**, the results that the contextual factor of authentic leadership directly affects employee resilience, indicate the importance of selecting, cultivating, and retaining leaders with authentic features. Therefore, construction companies can develop relational transparency, team-based simulation as a training tool, and structural empowerment to enhance authentic leadership, thereby improving employee psychological capital and work performance. **Second**, the verified organizational identification mediation within the authentic leadership-employee resilience relationship in the Chinese organizational context highlights the significance of building/maintaining employees’ identifications with their organizations by authentic leaders, as well as the important role organizational identification played in building, maintaining, and enhancing employee resilience. That being said, construction companies should create external favorable conditions for employees to identify with their organization by promoting organizational support, trust, and communication. **Third,** the finding that flow positively moderates the relationship between organizational identification and employee resilience, suggests the organization can provide enabling working conditions and develop mindful work performance enhancement interventions to enhance flow at work, which could contribute to a resilience development program. For example, construction engineering project companies can nudge employees’ flow through smart goal setting, motivating job characteristics, and crafting a to-do list. However, in terms of flow’s negative moderation effects on the links from authentic leadership to employee resilience and from authentic leadership to organizational identification, employees entering in flow may sometimes feel excessively effective and are often directed by these sensations if they lack information that might aid in judging the potentially risky situation, which may lead them to underestimate and take risks. While flow is a double-edged sword, preventing it is not an option due to the benefits of flow on job tasks; thus, senior managers can assist employees in rapidly gaining “experience,” for example, through risk education, practical risk training, and safety rules. To summarize, fostering employee resilience may have a favorable effect on the quality of work provided by employees in their organizations, and employees are recommended to strengthen their connections with organizations and leaders and armed with a combination of skills, capacity to adapt, knowledge, and other available capitals when responding to demanding challenges at work.

### Limitations and Future Research

Several limitations are noteworthy in the current study. First, the cross-sectional design confined to employees from this construction engineering project organization in China brings caution for causality as well as for generating findings to other populations. Therefore, ongoing research of longitudinal study via diary approach with time series cross-lagged analysis will disentangle their relationship to discover the more dynamic and causal inference between authentic leadership and employee resilience, taking organizational identification and flow into consideration. Future research will also welcome experimental research with cross-cultural data to dig into the relationship as proposed in the present work, as well as to examine the combined effect of the above factors on employee resilience with fuzzy set qualitative comparative analysis (fsQCA). Furthermore, the daily underlying mechanisms of the interactions among these state-based constructs still remain unknown. In order for the flow experience to possibly and effectively enhance organizational identification and shape authentic leadership and employee resilience at the within-person level, we welcome further research taking the above variables into consideration at the basis of both trait level and state level.

## Data Availability Statement

The original contributions presented in this study are included in the article/supplementary material, further inquiries can be directed to the corresponding authors.

## Author Contributions

YM, JM, and SL conceived the study. YM, YZ, JY, and MX developed the materials and gathered data. YM, YL, and SL conducted the analyses. YL, MX, QW, JY, SL, and YM prepared the manuscript. YM, YL, MX, JM, and MB revised the manuscript. All authors provided feedback to the revision and approved the publishing the final revised manuscript.

## Conflict of Interest

YZ was employed by the China Railway Construction Group Co., Ltd. The remaining authors declare that the research was conducted in the absence of any commercial or financial relationships that could be construed as a potential conflict of interest.

## Publisher’s Note

All claims expressed in this article are solely those of the authors and do not necessarily represent those of their affiliated organizations, or those of the publisher, the editors and the reviewers. Any product that may be evaluated in this article, or claim that may be made by its manufacturer, is not guaranteed or endorsed by the publisher.
